# Echo Preprocessing to Enhance SNR for 2D CS-Based ISAR Imaging Method

**DOI:** 10.3390/s18124409

**Published:** 2018-12-13

**Authors:** Zhiping Yin, Xinfei Lu, Weidong Chen

**Affiliations:** 1 National Key Laboratory of Advanced Display Technology, Academy of Photoelectric Technology, Hefei University of Technology, Hefei 230009, China; zpyin@hfut.edu.cn; 2 Key Laboratory of Electromagnetic Space Information, Chinese Academy of Sciences, University of Science and Technology of China, Hefei 230027, China; lxfei@mail.ustc.edu.cn

**Keywords:** ISAR, echo denoising, SNR enhancing, matrix optimization, 2D CS

## Abstract

A new CS-based inverse synthetic aperture radar (ISAR) imaging framework is proposed to enhance both the image performance and the robustness at a low SNR. An ISAR echo preprocessing method for enhancing the ISAR imaging quality of compressed sensing (CS) based algorithms is developed by implementing matched filtering, echo denoising and matrix optimization sequentially. After the preprocessing, the two-dimensional (2D) SL0 algorithm is applied to reconstruct an ISAR image in the range and cross-range plane through a series of 2D matrices using the 2D CS theory, rather than converting the 2D convex optimization problem to the one-dimensional (1D) problem in the image reconstruction process. The proposed preprocessing framework is verified by simulations and experiment. Simulations and experimental results show that the ISAR image obtained by the 2D sparse recovery algorithm with our proposed method has a better performance.

## 1. Introduction

In the synthetic aperture radar (SAR) and inverse synthetic aperture radar (ISAR) imaging systems, the range resolution is determined by the bandwidth of the transmitting signal. The larger the bandwidth is, the better the resolution will be [1]. However, a large signal bandwidth requires a massive amount of data and an expensive system. In addition, the cross-range resolution depends on the total rotation angle of the target during the observation time [1]. However, the range cell migration, which is usually caused by a wide rotation angle, greatly degrades the ISAR imaging quality. Hence, it is hard to achieve a high-resolution ISAR imaging in practice because the imaging quality is limited by a narrow bandwidth and a small aperture. 

In the last decade, a new signal sampling theory called compressed sensing (CS) [2] has been proposed to reduce the cost of data acquisition and storage and has been successfully applied to the optics and microwave imaging system [3,4]. The CS-based algorithms can reconstruct the sparse signals accurately from much less measurements than the ones mandated by Nyquist’s theorem, through solving an optimization problem with high probability [2]. In the ISAR imaging applications, the target of interest, such as aircrafts in the air or ships in the sea, can be proximately regarded as a sum of responses from prominent scattering centers in an almost clean background. Then, the number of strong scattering centers of the target is much less than that of the pixels in the image plane. This means that the radar targets can be considered sparse or compressible. Consequently, the CS is considered as a suitable method to deal with the ISAR image reconstruction from undersampled or sparse-aperture data. Besides, it has been shown that a high-resolution ISAR image can be achieved with a limited number of samples using the CS [5,6].

The CS has been successfully used in the SAR/ISAR to reduce the acquired data size, compensate for missing data and obtain high-resolution images, which cannot be achieved by using the traditional Fourier transform (FT) reconstruction method. However, the CS theory generally solves the one-dimensional (1D) problem. Although the two-dimensional (2D) ISAR data is usually respectively processed by range dimension and cross-range dimension for traditional imaging methods, it must be converted into a 1D vector for CS reconstruction. However, that increases the computational cost and memory consumption enormously. With the aim to reduce the computational cost of the conventional 1D CS-based algorithms, a few 2D sparse recovery algorithms based on the 2D CS, such as the two-dimensional iterative adaptive algorithm (2D IAA) [7], the two-dimensional sparse learning via iterative minimization (2D SLIM) [8], the two-dimensional fast iterative shrinkage-thresholding algorithm (2D-FISTA) [9] and the two-dimensional smoothed *l*_0_ norm algorithm (2D SL0) [10], were proposed. The smoothed *l*_0_ norm (SL0) approach was expanded into two dimensions, making the 2D smoothed *l*_0_ norm algorithm able to deal with the sparse reconstruction of 2D signals on dictionaries with separable atoms [10]. The high-resolution fully polarimetric ISAR images were obtained by using the 2D SL0 algorithm to solve the optimization problem constraint [5]. The 2D SL0 algorithm was applied directly to the 3D SAR based on the 2D signals [11]. The 2D SL0 algorithm was also utilized in passive ISAR imaging [12].

Most of the existing research about the performance improvement of CS in the ISAR was focused on reducing the computational cost and memory consumption or improving the performance of the CS-based algorithms by using complex constraint functions to describe the sparsity of signals in a better way. Nevertheless, as Candès et al. pointed out, the signal-to-noise ratio (SNR) and the mutual coherence of the observation matrix are the two most important factors that affect the performance of the CS-based methods [13,14]. In reference [15], it was shown that the performance of the CS-based algorithms seriously depends on the input SNR and it cannot work well at a low input SNR in the SAR imaging. The quality of an ISAR image reconstructed from a sparse aperture with limited measurements was discussed in reference [16] regarding the SNR, and a CS-based model with a robust performance with the decrease in the SNR was presented [16]. The CS-based ISAR imaging was improved to overcome strong noise and clutter by combining the coherent projectors and weighting with the CS optimization at low SNR [17]. The low-rank denoising operation was used to enhance the robustness of the ISAR sparse imaging based on the CS at a low SNR [18]. 

In the literature mentioned above, the improvement of computational efficiency and robustness at a low SNR was discussed in different paper. In this paper, a new CS-based ISAR imaging framework is presented to improve both the performance and the robustness at a low SNR. First, an echo preprocessing is implemented before the 2D CS recovery to improve the SNR of ISAR echo and to reduce the mutual coherence of the observation matrix. Then, the 2D SL0 algorithm is applied to reconstruct the ISAR image in range and cross-range plane through a series of 2D matrices using 2D CS theory, rather than converting the 2D convex optimization problem to the 1D problem in the process of image reconstruction. 

As the SNR and the mutual coherence of the observation matrix are the most important factors of CS-based methods, for the first time two methods are combined to improve the SNR. The SNR can be increased using the MF and echo denoising, but in that case, the mutual coherence of the observation matrix worsens due to the increased size of the observation matrix. Therefore, we use the observation matrix optimization method to improve the mutual coherence and to reduce the size of observation and the computation load of CS-based methods. Finally, we use the 2D SL0 as the CS-based method to verify the proposed signal preprocessing framework. In this work, we focus on the signal preprocessing framework which has not been used in other papers, and we use some classical methods because we focus on the preprocessing, not the classical methods modification. 

Notations used in this paper are as follows. Bold case letters are reserved for matrices. svd(A) denotes the singular value decomposition of matrix A. ${\left\|A\right\|}_{2}$ and ${\left\|A\right\|}_{F}$ are the *l*_2_ norm and the Frobenius norm of matrix A. ${\left(A\right)}^{H}$ denotes the conjugate transpose of matrix A. ${\left(A\right)}_{m,n}$ represents an element in the *m*th row and the *n*th column of matrix A. rank(A) is the rank of matrix A. diag(A) is a diagonal matrix having the same diagonal elements as matrix A. rand(m,n) is a function which generates a matrix with the size of m×n containing random elements.

## 2. ISAR Model

Following the Born approximation, the received acquisition ISAR echo at the *m*th frequency and the *n*th aspect angle can be expressed as:

(1) $$Y\left(m,n\right)=\iint_{S}\sigma \left(x,y\right)e^{-j4\pi \left(\frac{f_{m}x}{c}+\frac{\theta_{n}y}{\lambda_{c}}\right)}dxdy+N\left(m,n\right)$$

where σ(x,y) denotes the backscattering coefficient of the point scatter located at (x,y), *S* is the imaging area. c and $\lambda_{c}$ represent the wave velocity and wavelength, respectively. $f_{m}$ is the *m*th signal frequency, and $\theta_{n}$ is the *n*th observation angle. N(m,n) is the noise with Gaussian distribution [19]. 

The grids of the imaging plane can be refined by choosing P>M for *x* dimension and Q>N for *y* dimension. Then (1) can be expressed in a matrix form

(2) $$Y=A_{x}\sigma A_{y}^{H}+N$$

where Y is the acquisition echo matrix with the size of M×N, σ is the interested ISAR image with the size of P×Q, $A_{x}$ with the size of M×P and $A_{y}^{H}$ with the size of Q×N denote the observation matrices in the range and cross-range directions, respectively, and they are defined as

(3) $${\left(A_{x}\right)}_{m,p}=e^{-j\frac{4\pi }{c}f_{m}x_{p}},\;{\left(A_{y}^{H}\right)}_{q,n}=e^{-j\frac{4\pi }{c}\theta_{n}y_{q}}$$

We are supposing that K<<min(P,Q), which means that only a few strong scattering centers occupy the whole image plane, so the image of the target is sparse. The CS-based methods can accurately reconstruct σ from the limited measurements Y by solving an optimization problem with a high probability.

The echo preprocessing can be used to improve the SNR and observation matrix property. As we mentioned before, the SNR of the ISAR echo and the mutual coherence of the observation matrix are the two key factors which have a significant influence on the imaging quality of the CS-based methods. Therefore, we propose a novel CS-based ISAR imaging framework to enhance the SNR of echo matrix and improve the property of the observation matrix. The proposed framework is based on the following four steps.

## 3. Step 1: Matched Filtering

It is well known that the matched filtering (MF) is an optimal linear filter for maximizing the SNR in the presence of additive stochastic noise. Therefore, the MF is used to maximize the SNR of echo matrix Y. The MF used in this work represents the constructing of matching vectors. The matching matrix is formed by all the matching vectors. The matching vectors are constructed using the same grids as σ. Thus, the matching matrix is the same as $A_{x}$ and $A_{y}$. The matching process can be represented by a matrix operation. The echo after the MF process can be written as

(4) $$\tilde{Y}=A_{x}^{H}YA_{y}={\tilde{A}}_{x}\sigma {\tilde{A}}_{y}^{H}+\tilde{N}$$

where ${\tilde{A}}_{x}=A_{x}^{H}A_{x}$, ${\tilde{A}}_{y}=A_{y}^{H}A_{y}$, and $\tilde{N}=A_{x}^{H}NA_{y}$.

Where ${\tilde{A}}_{x}$ with the size of P×P and ${\tilde{A}}_{y}$ with the size of Q×Q denote the new observation matrix. The size of the filtered echo matrix $\tilde{Y}$ becomes P×Q, which is usually much larger than the size of the raw echo matrix Y (M×N), which further means that the mutual coherence of the new observation matrices ${\tilde{A}}_{x}$ and ${\tilde{A}}_{y}$ will increase because the size of the new observation matrices is much larger compared with the size of $A_{x}$ and $A_{y}$. Besides, the noise cannot be eliminated by the MF completely. The SNR of echo is usually improved obviously after the MF, but in some cases, it is still not high enough to meet requirements for relative SNR of the CS-based ISAR imaging method. Therefore, the low-rank denoising operation is used to further enhance the reliable performance of the CS-based ISAR imaging method at a low SNR.

## 4. Step 2: Echo Denoising

To enhance the SNR of echo further, the low-rank property of echo matrix combined with echo denoising algorithm given by (5) is proposed.

(5) $$\bar{Y}=Denoise(\tilde{Y})$$

As $rank({\tilde{A}}_{x}\sigma {\tilde{A}}_{y}^{H})\leq \min\left(rank\left({\tilde{A}}_{x}\right),\;rank\left(\sigma \right),\;rank\left({\tilde{A}}_{y}^{H}\right)\right)$, then it holds that $rank({\tilde{A}}_{x}\sigma {\tilde{A}}_{y}^{H})\leq rank\left(\sigma \right)$. Due to $rank(\bar{Y})\leq K<<\min(P,Q)$, matrix $\bar{Y}$ satisfies the low-rank property, and it can be solved by the weighted constrained optimization problem [20]:

(6) $$\min_{\bar{Y}}{\sum_{i}\omega_{i}\gamma_{i}(\bar{Y})s.t.\left\|\tilde{Y}-\bar{Y}\right\|}_{F}^{2}<\delta$$

where $\gamma_{i}(\bar{Y})$ is the *i*th singular value of matrix $\bar{Y}$, and $\omega_{i}$ is the weight of the *i*th singular value. The inexact augmented Lagrange multiplier method (inexact ALM) [21] is applied to solve the matrix denoising problem as described in Algorithm 1.

**Algorithm 1.** Echo denoising via the inexact ALM method**Initialization:**${\bar{Y}}_{0}=\tilde{Y}$, $Z_{0}={\tilde{Y}/\left\|\tilde{Y}\right\|}_{2}$, $\mu_{0}>0$, ρ>1, ε>0, l=0 (1) $\left(U,S,V\right)=svd\left({\bar{Y}}_{l}\right)$ (2) $W=\frac{1}{diag\left(S\right)+\varepsilon }$ (3) $\left(U,S,V\right)=svd\left(\tilde{Y}+\frac{Z_{l}}{\mu_{l}}\right)$ (4) ${\bar{Y}}_{l+1}=US_{\frac{W}{\mu_{l}}}\left[S\right]V$ (5) $Z_{l+1}=Z_{l}+\mu_{l}\left(\tilde{Y}-{\bar{Y}}_{l}\right)$ (6) $\mu_{l+1}=\rho \mu_{l}$ (7) l=l+1Stop after several iterations or do until ${\left\|{\bar{Y}}_{l}-{\bar{Y}}_{l-1}\right\|}_{F}<\varepsilon$
**Output:**
$\bar{Y}={\bar{Y}}_{l}$


Where $S_{\tau }(x)$ is the soft-thresholding operator defined as:

(7) $$S_{\tau }(x)=\{\begin{array}{cc} x-\tau , & if\;x>\tau \\ 0, & otherwise \end{array}$$

We apply the denoising algorithm on $\tilde{Y}$ instead of on Y because of the following two reasons: (1) The denoising algorithm is sensitive to the low-rank property of the matrix to be recovered. The size of $\tilde{Y}$ is P×Q which is much larger than the size of Y, but both of their rank are rank(σ). So, matrix $\tilde{Y}$ has better low-rank property than matrix Y. (2) The denoising algorithm cannot be applied at a very low SNR and the SNR of matrix $\tilde{Y}$ is much higher than that of matrix Y due to the operation of MF.

It should be noted that the denoising algorithm cannot suppress noise completely. Namely, it can only remove a part of the noise which is independent of the echo signal.

## 5. Step 3: Matrix Optimization

Taking into the consideration the above-mentioned responses, the mutual coherence of ${\tilde{A}}_{x}$ and ${\tilde{A}}_{y}$ is larger than that of $A_{x}$ and $A_{y}$. Therefore, in order to decrease the coherence, the matrix optimization algorithm combining the shrinkage-based algorithm [22] with the gradient-based alternating minimization approach [23] is used, and it is given in Algorithm 2.

**Algorithm 2.** Matrix optimization**Initialization:**$\Phi_{x}=rand(M^{\prime },P)$, $\left(M^{\prime }<M<P\right)$, $H=I_{P\times P}$, γ>0, β>0for l=1 to L do(1) $G={\left(\Phi_{x}{\tilde{A}}_{x}\right)}^{H}\left(\Phi_{x}{\tilde{A}}_{x}\right)$(2) H=(4/π)γarctan(G)(3) $\Phi_{x}=\Phi_{x}-\beta \Phi_{x}{\tilde{A}}_{x}\left(G-H\right){\tilde{A}}_{x}^{H}$end**Output**: $\Phi_{x}$

The same optimization operation is performed on ${\tilde{A}}_{y}$. After optimization of ${\tilde{A}}_{x}$ and ${\tilde{A}}_{y}$, the echo is given by

(8) $$\hat{Y}=\Phi_{x}\bar{Y}\Phi_{y}^{H}={\hat{A}}_{x}\sigma {\hat{A}}_{y}^{H}+\hat{N}$$

where, ${\hat{A}}_{x}=\Phi_{x}A_{x}^{H}A_{x}$, and ${\hat{A}}_{y}=\Phi_{y}A_{y}^{H}A_{y}$. 

After matrix optimization, the size of the observation matrix is reduced, and the mutual coherence is improved. Therefore, the imaging performance of the CS-based algorithm is improved, and the computation cost of the CS-based algorithm is reduced by using a smaller size observation matrix.

## 6. Step 4: Imaging Using 2D SL0

The size of matrix after the preprocessing conducted to improve the SNR is still large, so the computational cost and memory consumption will be enormous if the 1D CS algorithm is used to recover the backscattering coefficient matrix σ. Therefore, the 2D SL0 algorithm is used to reconstruct the ISAR image to reduce the computational cost [10]. Considering the noise, the interested 2D backscattering coefficient matrix σ can be obtained by solving (9)

(9) $$\min_{\sigma }{\left\|\sigma \right\|}_{0}\;s.t.\;{\left\|\hat{Y}-{\hat{A}}_{x}\sigma {\hat{A}}_{y}^{H}\right\|}_{F}^{2}\leq \varepsilon$$

The key point of the 2D SL0 is that it uses a continuous Gaussian function to approximate the $l_{0}$ norm of the signal. Thus, ${\left\|\sigma \right\|}_{0}$ in (9) can be approximated as follows:

(10) $${\left\|\sigma \right\|}_{0}\approx PQ-\sum_{p=1}^{P}\sum_{q=1}^{Q}\exp\left[-\frac{{\left|\sigma_{p,q}\right|}^{2}}{2\delta^{2}}\right]\;\mathrm{when}\;\delta \to 0$$

where ${\left\|\sigma \right\|}_{0}$ denotes the number of non-zero components in σ, and ε is a small constant, which is bounded by the noise level. Then, a projective steepest descent optimal approach can be used to find the minimum value of ${\left\|\sigma \right\|}_{0}$, i.e., the sparsest solution of (10), which makes this algorithm have high computational efficiency. The details of this algorithm can be found in reference [10]. 

## 7. Simulations Results and Discussion

To evaluate the performance of the proposed CS-based ISAR imaging method regarding the SNR of an original echo, the following simulations were conducted. The simulations parameters were shown as follows: $\lambda_{c}=0.01\;m$, $f_{m}=0∼2\;\mathrm{GHz}$, $\theta_{n}=0∼5{}^{\circ}$, M=N=100, P=Q=200, K=10. The 2D SL0 was chosen as the sparse recovery algorithm. The SNR was defined by (11), and $Y_{n}$ and $Y_{p}$ denoted the echo matrices with and without the noise, respectively.

(11) $$\mathrm{SNR}=20\times \log_{10}\left(\frac{{\left\|Y_{p}\right\|}_{F}}{{\left\|Y_{n}-Y_{p}\right\|}_{F}}\right)$$

**Figure 1:**
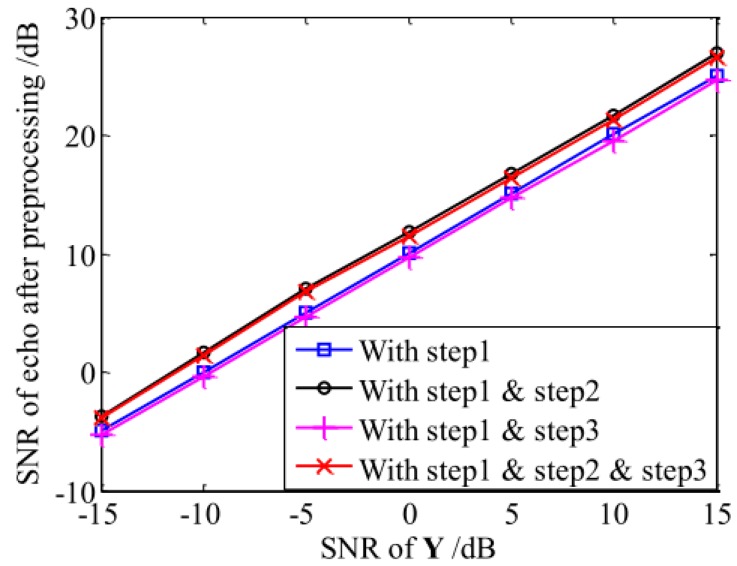
The SNR after applying different preprocessing methods to the original echo.

The SNR of the echo after applying different preprocessing methods in the Monte Carlo simulation for 200 times is presented in Figure 1. In Figure 1, it can be seen that the MF and the denoising algorithm improved the SNR of the echo. But, as already mentioned, noise could not be suppressed completely. 

The *t*-averaged mutual coherence [14] was used to measure the property of the observation matrix, and the obtained result is shown in Table 1 (*t* = 0.1). The mutual coherence of $\tilde{A}$ was larger than that of A because of the increased matrix size. Also, the proposed matrix optimization algorithm reduced the mutual coherence, which is in accordance with the above analysis.

To measure the performance of the images recovered by different preprocessing methods, the target-to-background ratio (TBR) defined by (12) was used.

(12) $$TBR=10\times \log_{10}\left(\sum_{(p,q)\in R_{T}}\left|I{\left(p,q\right)}^{2}\right|/{\sum_{(p,q)\in R_{B}}\left|I(p,q)\right|}^{2}\right)$$

where *I* denotes the recovered ISAR image, and $R_{T}$ and $R_{B}$ are the target and background regions, respectively. It should be noted that high TBR indicates a high ratio of target energy to noise energy in the recovered image. 

**Figure 2:**
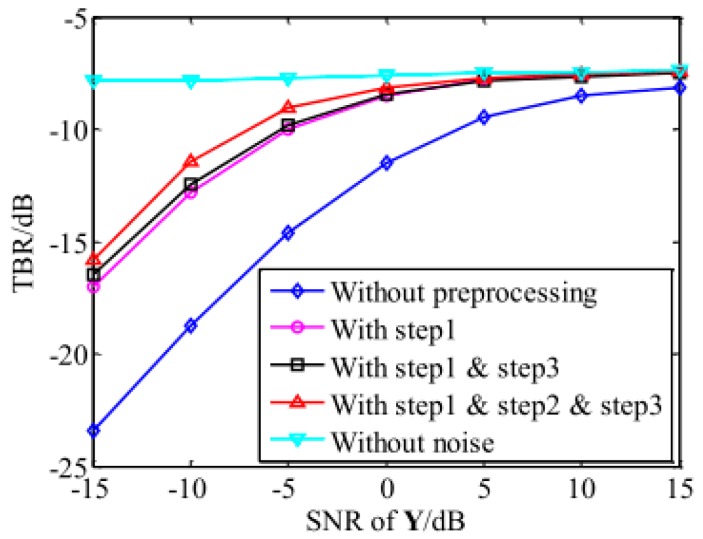
The TBR of the recovered images against the echo SNR.

The TBR comparison of the ISAR imaging results obtained by different echo preprocessing methods is presented in Figure 2. It can be observed that when the SNR increased, the TBR also increased and gradually approached that without noise. Besides, each step of the proposed method improved the performance. According to the obtained results, the proposed echo preprocessing method achieved a much better TBR than the traditional imaging technique without preprocessing.

## 8. Experimental Results and Discussion

**Figure 3:**
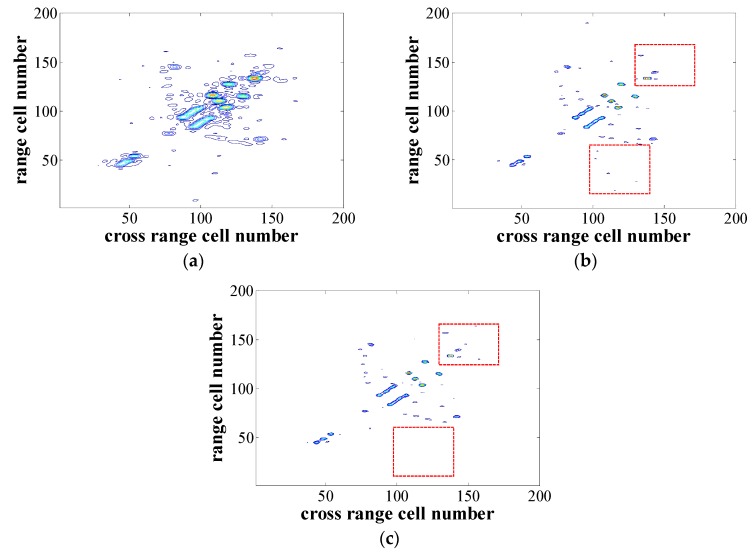
Imaging results. (**a**) Imaging result of the MF. (**b**) Imaging result of the 2D SL0 without echo preprocessing. (**c**) Imaging result of the 2D SL0 with the proposed echo preprocessing.

The quasi-real data of an airplane “B-727” provided by the U.S. Naval Research Laboratory, which can be found on the website http://airborne.nrl.navy.mil/~vchen/tftsa.html, was used to test the feasibility and performance of the proposed echo preprocessing method. The radar system model parameters are given in Table 2. The number of points in the range domain was 64, and 32 pulses were collected. The additive noise follows a complex white Gaussian distribution and the SNR was 5 dB. In the experiment, the 2D SL0 was used for the ISAR imaging. The results obtained by different processing methods are shown in Figure 3. It can be found that the resolution of the image obtained by MF method in Figure 3a was low and along with the high sidelobes. On the other hand, the image obtained by the CS-based method in Figure 3b,c had a higher resolution. However, the recovered result obtained by the proposed preprocessing method in Figure 3c had better performance in the regions marked by the red rectangles compared with the result obtained by the 2D SL0 method without echo preprocessing in Figure 3b, which reduced the false scattering points, and the loss of weak scattering points could be seen clearly.

## 9. Conclusions

The proposed preprocessing method improves the SNR of the ISAR echo by using the matched filtering and echo denoising technique, and optimizes the property of the observation matrix by reducing the mutual coherence of the observation matrix via matrix optimization. The 2D SL0 method is utilized instead of the 1D CS-based ISAR imaging method to reduce the cost and computation burden. The proposed preprocessing method was verified by the simulations and experiment. The simulations and experimental results showed that the proposed method improved the ISAR imagery quality and reduced the required SNR of the CS-based methods. Both of these improvements are helpful to radar identification, recognition and classification. 

## Figures and Tables

**Table 1:** *t*-Averaged mutual coherence.

	A	$\tilde{A}$	$\hat{A}$
$\mu_{t}(x)$	0.3547	0.3601	0.2082
$\mu_{t}(y)$	0.3321	0.3380	0.1923

**Table 2:** System model parameters for data “B-727”.

Parameter	Value
Carrier frequency	9 GHz
Bandwidth	150 MHz
Pulse repetition Interval (PRI)	3.2 ms
